# Onset and progression of postmortem histological changes in the kidneys of RccHan^TM^:WIST rats

**DOI:** 10.3389/fvets.2025.1578579

**Published:** 2025-05-09

**Authors:** Ricardo de Miguel, Raquel Vallejo, Kristel Kegler, Robert Kreutzer, Francisco José Mayoral, Yoshimasa Okazaki, Paula Ortega, Laura Polledo, Tanja Razinger, Olivia Kristina Richard, Raúl Sanchez, Nils Warfving, Anna Domènech, Klaus Weber

**Affiliations:** ^1^AnaPath Services GmbH, Liestal, Switzerland; ^2^AnaPath Research S.A.U., Barcelona, Spain

**Keywords:** autolysis, postmortem, rat, artifact, delayed fixation, decomposition, kidney, histology

## Abstract

**Introduction:**

Death initiates a cascade of physiological and biochemical alterations in organs and tissues, resulting in microscopic changes that challenge the histopathological evaluation. The aim of this study was to compile and illustrate the microscopic changes in the kidneys of rats subjected to delayed postmortem fixation. It also scrutinizes the influence of exsanguination, cooling methods and air circulation on the initiation and progression of these alterations.

**Methods:**

Twenty-four Wistar Han outbred rats (RccHan^TM^:WIST) were sacrificed and stored either at room temperature (18–22°C; half of the carcasses were exsanguinated after sacrifice) or under refrigeration (2–4°C). Necropsies were conducted at different time points postmortem (i.e., 0.5, 1, 4, 8, 12, 24, 36, 48 h for carcasses stored at room temperature, and 7 and 14 days for carcasses stored under refrigeration). Kidney sections underwent simultaneous digital evaluation by 14 pathologists until a consensus was reached on the key findings, terminology, and intensity levels.

**Results:**

When stored at room temperature, the first changes were seen after 4 h, and involved distal convoluted tubules and inner stripe of the outer medulla. After 8 h, all structures except glomeruli were affected. Alterations were similar in quality and intensity after 36 h as after 48 h. Exsanguination delayed the onset of postmortem changes and slightly decreased their overall severity at any given timepoint. The nature of the changes under refrigeration was like those alterations noted in animals stored at room temperature. The intensity of postmortem changes observed after 7 and 14 days under refrigeration was similar to those recorded after 48 h at room temperature. No clear differences were observed between animals stored in a closed plastic bag and animals stored in a perforated cardboard box to allow air circulation.

**Conclusion:**

This work elucidates the onset and progression of postmortem changes in the kidneys of Wistar Han rats, offering insights to accurately differentiate them from real changes and enhance histopathological evaluation.

## 1 Introduction

Systemic animal death is defined as the cessation of the brain, heart, and lungs vital functions. Death implies the halt of blood flow, which triggers a sequence of physical and chemical alterations in tissues and cells leading to postmortem changes (1). These changes manifest as distinct phenomena, including algores mortis, livores mortis, rigores mortis, desiccation, decomposition, and mummification (2, 3). They often arise simultaneously and are influenced by various internal and external factors, such as temperature, humidity, oxygen levels, and invertebrate activity (3, 4).

Decomposition of the body arises from two primary processes: autolysis and putrefaction. Autolysis involves the self-digestion of cells driven by their own enzymes, which are released and activated due to disrupted cellular homeostasis and compromised cell membrane integrity. Activation of enzymes is the consequence of exhaustion of ATP and pH drop caused by the oxygen deprivation in cells, the associated failure of oxidative phosphorylation, and the counterbalance activation of anerobic glycolysis. Moreover, in some organs autolysis creates ideal conditions for bacterial proliferation leading to putrefaction (3).

The rat kidney has a unique unipapillary structure that can be divided into distinct compartments including the cortex, outer stripe of the outer medulla (OSOM), inner stripe of the outer medulla (ISOM), inner medulla, papilla and pelvis (5–8). These anatomic subdivisions contain specific portions of the nephron and show different histological features and cellular composition, which contribute to its physiological functions (7, 8). The renal cortex contains glomeruli and proximal and distal convoluted tubules (PCT and DCT), where essential filtration, reabsorption, and secretion processes occur. Below the cortex lies the OSOM, consisting mainly of the pars recta of the proximal tubules, thick ascending limb of Henle's loops and collecting ducts, which play a crucial role in maintaining salt and water balance. The ISOM, deeper still, contains parts of the thin descendent and thick ascending limbs of Henle's loops, essential for countercurrent multiplication and urine concentration (8). The inner medulla is primarily composed of collecting ducts and thin ascending and thin descending limbs of Henle's loops, further concentrating urine through water reabsorption facilitated by the osmotic gradient. The papilla, extending into the renal pelvis, serves as the final point for urine collection, where fluid flows into the pelvis before entering the ureter.

Autolysis and putrefaction are widely known artifacts of the kidneys in unproperly fixed specimens. These postmortem changes betrays forensic, diagnostic, and toxicologic pathologists during routine histopathological evaluation because these can either hinder the identification of some lesions or, alternatively, mimic the histological appearance of real findings favoring misdiagnoses (9, 10). However, there is scarce information to properly identify histological postmortem changes. This highlights the need for a comprehensive description and illustration of these changes to avoid the distortion or misidentification of real findings (3). The aim of this study was to systematically compile and illustrate the microscopic changes in rat kidneys subsequent to delayed postmortem fixation. Furthermore, we aimed to analyze the potential effect of exsanguination, cooling, and air ventilation on the onset and progression of these findings.

## 2 Materials and methods

### 2.1 Study design

All animals employed in the present study were surplus naïve animals from regulatory studies approved by the Ethical Committee of the Generalitat de Catalunya (Departament d'Acció Climàtica, Alimentació i Agenda Rural) and licensed under ref. 10832. Requirements of the Spanish Policy for Animal Protection (RED118/2021 and RED1386/2018) and the European Union Directive 2010/63 on the protection of experimental animals were always fulfilled.

The study design was previously described (11). In total, 24 Wistar Han outbred rats (RccHan^®^:WIST) were sacrificed by intraperitoneal overdose of phenobarbital sodium (Dolethal^®^, Vetoquinol). When applicable, and mimicking inhouse standard protocols in regulatory studies, exsanguination was performed immediately after death by bilateral section of brachial plexus and abdominal aorta after ventral cavity exposure, extravasated blood was aspirated using a suction device until bleeding stopped.

Sixteen carcasses were stored uncovered inside cages under controlled room temperature (18–22°C) to mimic the conditions of animal housing rooms. Of these, eight carcasses had been exsanguinated whereas the other eight carcasses remained non-exsanguinated. Rats were necropsied at different timepoints after euthanasia (i.e., 0.5, 1, 4, 8, 12, 24, 36, and 48 h). At each timepoint, an exsanguinated and a non-exsanguinated animal were necropsied (Supplementary Table 1).

Eight additional non-exsanguinated carcasses were stored in a refrigerator (2–4°C). Of these, four carcasses were placed in a perforated cardboard box and the other four carcasses in an opaque plastic bag, thereby mimicking long-term storage conditions with and without air circulation, respectively. These animals were necropsied 7 and 14 days after euthanasia. At each timepoint, two animals (one male and one female) stored in the cardboard box and two (one male and one female) stored in the plastic bag were necropsied. The distribution of sexes for the different study conditions are provided in Supplementary Table 1.

### 2.2. Histoprocessing and histopathology evaluation

Kidneys were sampled at necropsy and fixed by immersion in 10% neutral phosphate-buffered formalin for 48 to 72 h, maintaining an organ to formalin ratio of 1:20. At trimming, transversal sections were performed, embedded in paraffin, cut at a nominal thickness of 2–4 μm, and stained with hematoxylin-eosin. Evaluation included the following anatomical locations: Renal cortex; OSOM; ISOM; Inner medulla; and Renal pelvis and papilla.

Samples were scanned with a 20× objective (200× magnification) by an Olympus Slideview VS200 slide scanner coupled to a VS-264C camera. Digital slides were analyzed concurrently by 14 pathologists until a shared consensus was reached on the most relevant postmortem histological changes, their intensity degree, and the terminology employed. The team of pathologists was composed of professionals with different levels of expertise and educational backgrounds including ECVP, JCVP or JSTP board-certified pathologists.

Descriptive terminology, avoiding the use of specific diagnostic terms, was systematically applied to characterize the main histological changes observed. Postmortem changes were scored from 0 to 3 (i.e., 0 = absent to minimal; 1 = mild; 2 = moderate; 3 = marked) based on their distribution, extension, and intensity degree.

## 3 Results

### 3.1 Terminology

The terms employed to describe the postmortem alterations observed in the kidney are displayed in Table 1. The terminology employed was previously used and described in previous works on different organs (11). A concise explanation of each term and alternative terms employed in the literature are included. The following abbreviations were employed to refer to different renal structures: PCT (Proximal convoluted tubules), DCT (Proximal convoluted tubules), OSOM (Outer stripe of the outer medulla), ISOM (Inner stripe of the outer medulla).

**Table 1:** Description of postmortem histological findings in the kidneys of Wistar Han rats and the cell type or anatomical region affected.

**Finding**	**Location**	**Description**
Nuclear shrinkage	Cortex: Glomeruli, PCT and DCT OSOM and ISOM. Inner medulla	Decreased nuclear size. Associated with chromatin condensation at earlier timepoints and progressive nuclear fading at later timepoints.
Chromatin condensation	Cortex: Glomeruli, PCT and DCT OSOM and ISOM. Inner medulla	Chromatin clumping and agglomeration leading to highly compacted and intensely stained nuclei. Described in other publications as “Clumping of chromatin” or “Pyknosis” (10, 17, 30).
Nuclear fading	Cortex: Glomeruli, PCT and DCT OSOM and ISOM. Inner medulla	Decreased nuclear basophilia and less discernible nucleus. Described in other publications as “Loss of nuclear basophilic staining,” “Unstained nuclei” or “Dissolution of nuclear chromatin” (10, 12, 18, 30).
Homogenization of the mesangium	Cortex: Glomeruli	Homogeneous staining of the mesangium with loss of cytoplasmic and extracellular matrix details.
Granular cellular debris	Cortex: Glomeruli	Presence of cellular debris mainly composed of flocculent cytoplasmic material in the glomerular space. Described similarly in some publications (38) and as “Cytoplasmic debris” in others (17).
Cellular swelling	Cortex: PCT and DCT OSOM and ISOM. Inner medulla	Increase in cell volume associated to an increase in the cytoplasmic content. Described similarly in some publications (17, 38) and as “Cytoplasmic vacuolation” in others (12).
Cytoplasmic dissolution	Cortex: PCT and DCT OSOM and ISOM. Inner medulla	Increased granulation and/or flocculent or ground-glass appearance of the cytoplasm. Associated with decreased staining affinity and progressive cytoplasmic shrinkage and loss. Described as “Solution of protoplasm” or “Degenerative cytoplasmic changes” in other publications (12, 38).
Cellular rupture	Cortex: PCT and DCT. ISOM.	Breakdown of the cell with fragmentation of the plasma membrane, loss of cell borders and release of cytoplasmic and nuclear contents. Described as “Loss of cell membrane” in other publications (17).
Cellular detachment	Inner medulla.	Separation of epithelial lining cells from the underlying basement membrane and adjacent structures without separation from adjacent cells. Described identically in some publications (10).
Sloughing	Cortex: PCT and DCT. ISOM	Shedding or casting off cell remnants into a lumen. Described as “Desquamation” in other publications (17).
Cellular shrinkage	Inner medulla.	Decreased cellular size.
Coalescence of epithelial cells	Cortex: PCT	Loss of cell borders and continuity among cytoplasmic contents of cells
Protrusion of proximal tubule	Cortex: Glomeruli	Presence of proximal tubule within the glomerular space.

PCT, Proximal convoluted tubules; DCT, Distal convoluted tubules; OSOM, Outer stripe of the outer medulla; ISOM, Inner stripe of the outer medulla.

### 3.2 Onset and progression of postmortem histological changes in non-exsanguinated rats, stored at room temperature

**Figure 1:**
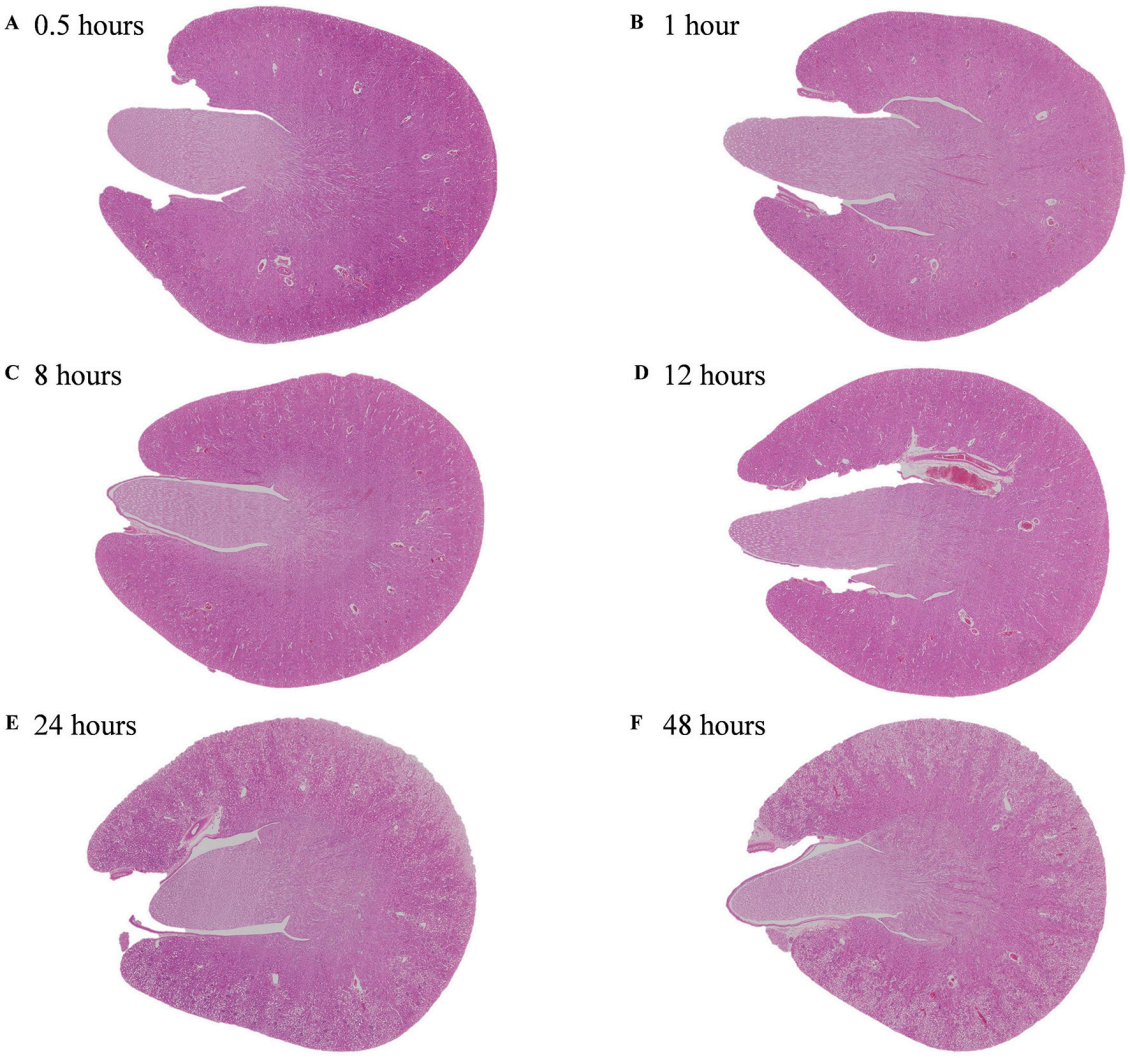
Overview images of the kidney of non-exsanguinated outbred RccHan^TM^: WIST rats at different timepoints after a delayed postmortem fixation. Carcass stored at room temperature (18–22°C). Hematoxylin-eosin stain. **(A)** 0.5 h. **(B)** 1 h. **(C)** 8 h. **(D)** 12 h. **(E)** 24 h. **(F)** 48 h.

Postmortem findings in the kidneys showed a different onset and progression in each of the anatomical compartments (Table 2). The Cortex and the ISOM were the most affected compartments while the OSOM and inner medulla were the least affected ones. Additionally, a sharp demarcation between the medullary rays of the OSOM and the cortical labyrinths of the tubules at later timepoints was evidenced at low magnification (Figure 1).

**Table 2:** Summarized postmortem histological changes in the kidney of non-exsanguinated outbred RccHan^TM^: WIST rats stored at room temperature (18–22°C) and necropsied at different timepoints after death.

	**Time after death**							
	**0.5 h**	**1 h**	**4 h**	**8 h**	**12 h**	**24 h**	**36 h**	**48 h**
**CORTEX—Glomeruli**								
Chromatin condensation and nuclear shrinkage	–	–	–	–	1	1	2^a^	2^a^
Homogenisation of mesangium	–	–	–	–	2	1	2	2
Granular cellular debris	–	–	–	–	1	2	2	2^b^
*Average*	0.00	0.00	0.00	0.00	1.33	1.33	2.00	2.00
**CORTEX—Proximal convoluted tubule (PCT)**								
Chromatin condensation and nuclear shrinkage	–	–	1	1	1^a^	2^a^	3^a^	3^a^
Cellular swelling and cytoplasmic dissolution	–	–	–	1	1	2	N/Q	N/Q
Cellular rupture and sloughing	–	–	–	–	1^c^	2^c^	3^c^	3^c^
Coalescence of epithelia	–	–	–	–	–	–	2	3
*Average*	0.00	0.00	0.25	0.50	0.75	1.50	2.75	3.00
**CORTEX—Distal convoluted tubule (DCT)**								
Chromatin condensation and nuclear shrinkage	–	–	2	2	2	3	3	3
Cellular swelling and Cytopl. dissolution	–	–	1	2	3	N/Q	N/Q	N/Q
Cellular rupture and sloughing	–	–	–	1	2	3	3	3
*Average*	0.00	0.00	1.00	1.67	2.33	3.00	3.00	3.00
**Outer stripe outer medulla (OSOM)**								
Chromatin condensation and nuclear shrinkage	–	–	1	1	2	2	2^a^	2^a^
Cellular swelling and Cytopl. dissolution	–	–	–	–	1	2	3	3
*Average*	0.00	0.00	0.50	0.50	1.50	2.00	2.50	2.50
**Inner stripe outer medulla (ISOM)**								
Chromatin condensation and nuclear shrinkage	–	–	3	3	3	3	3	3
Cellular swelling and Cytopl. dissolution	–	–	2^d^	3^d^	3^d^	3^d^	3	3
Cellular rupture and sloughing	–	–	1	1	1	2	3	3
*Average*	0.00	0.00	2.00	2.33	2.33	2.67	3.00	3.00
**Inner medulla**								
Cellular detachment	–	–	–	1	2	2	3	3
Cellular shrinkage	–	–	–	–	–	1	2	2
*Average*	0.00	0.00	0.00	0.50	1.00	1.50	2.50	2.50
**Pelvis and papilla**								
Urothelium detachment and sloughing	–	–	–	1	1	2	3	3

a Associated with nuclear fading;

b Protrusion of proximal tubule;

c Ass. with lumen debris;

d More pronounced basally.

**Figure 2:**
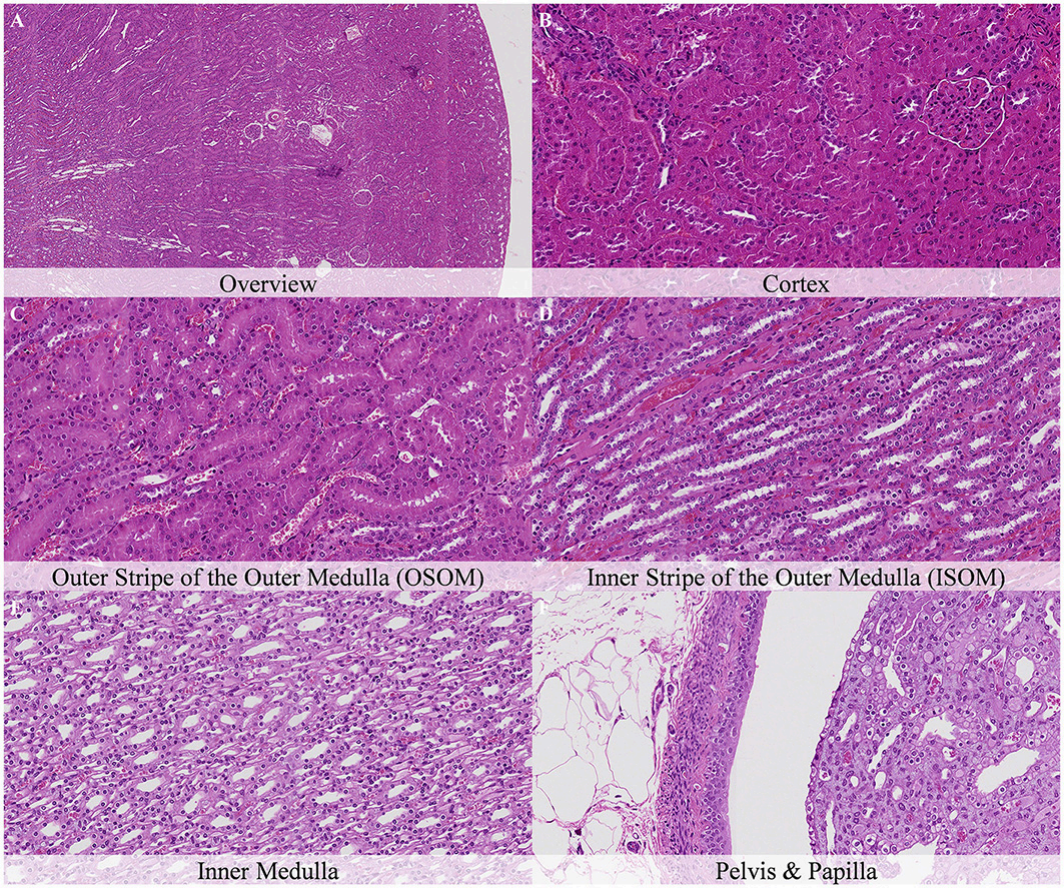
No postmortem microscopic changes in the kidney of non-exsanguinated outbred RccHan^TM^: WIST rats fixed 30 min after death. Carcass stored at room temperature (18–22°C). Hematoxylin-eosin stain. **(A)** Renal cortex, 20× magnification. **(B)** Renal cortex, 100× magnification. **(C)** Outer stripe of the outer medulla (OSOM), 100× magnification. **(D)** Inner stripe of the outer medulla (ISOM), 100× magnification. **(E)** Inner medulla, 100× magnification. **(F)** Pelvis and papilla, 100× magnification.

**Figure 3:**
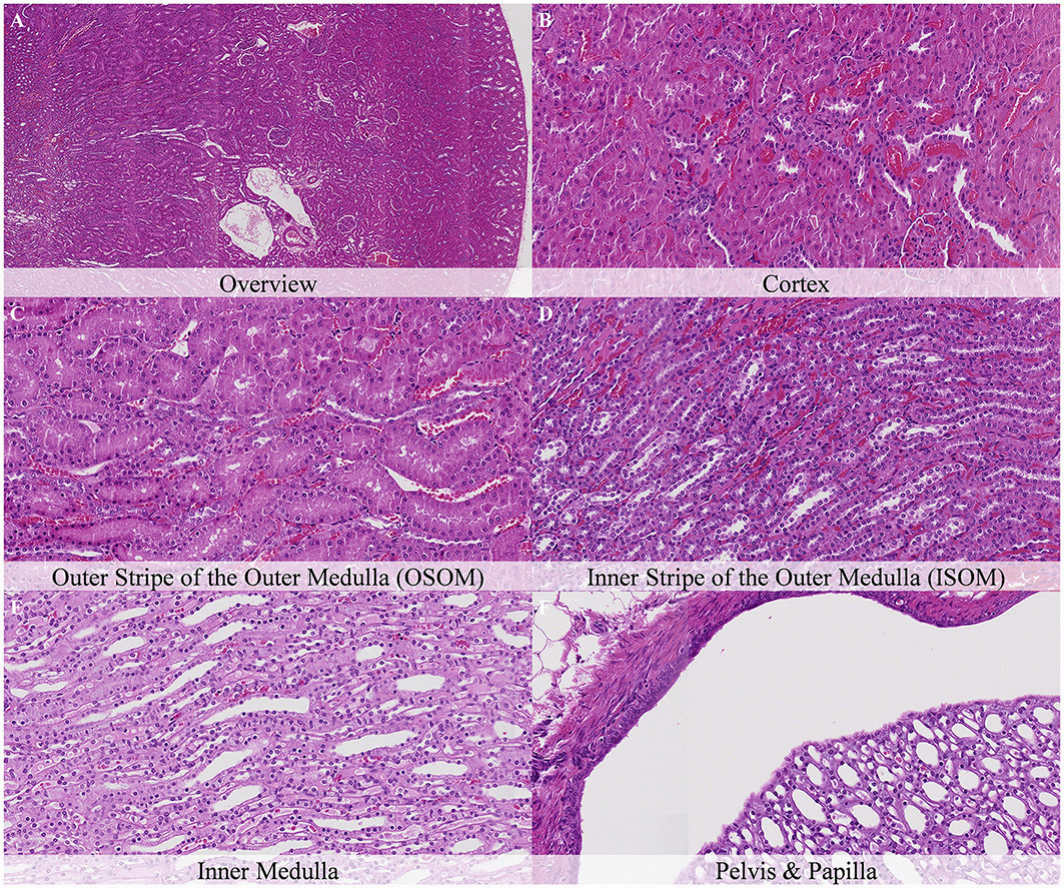
No postmortem microscopic changes in the kidney of non-exsanguinated outbred RccHan^TM^: WIST rats fixed 1 h after death. Carcass stored at room temperature (18–22°C). Hematoxylin-eosin stain. **(A)** Renal cortex, 20× magnification. **(B)** Renal cortex, 100× magnification. **(C)** Outer stripe of the outer medulla (OSOM), 100× magnification. **(D)** Inner stripe of the outer medulla (ISOM), 100× magnification. **(E)** Inner medulla, 100× magnification. **(F)** Pelvis and papilla, 100× magnification.

No postmortem findings were recorded 30 min and 1 h after death (Figures 2, 3).

**Figure 4:**
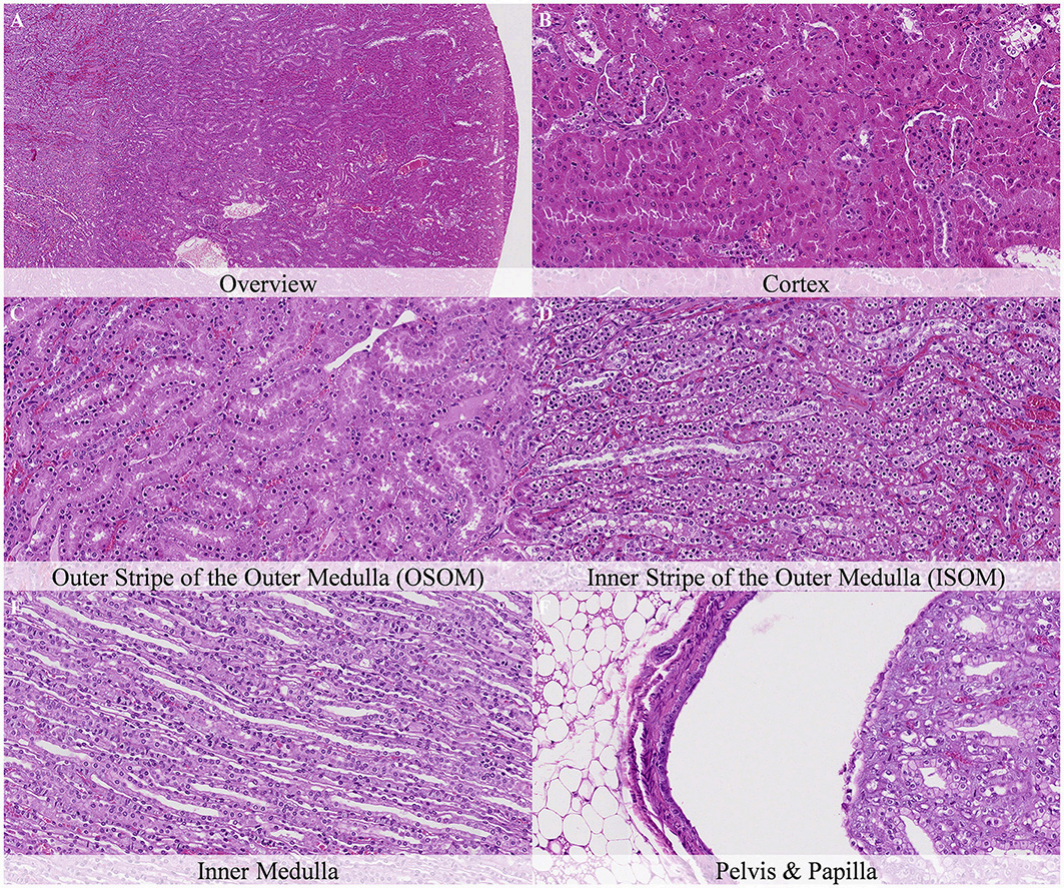
Postmortem microscopic changes in the kidney of non-exsanguinated outbred RccHan^TM^: WIST rats fixed 4 h after death. Carcass stored at room temperature (18–22°C). Hematoxylin-eosin stain. **(A)** Renal cortex, 20× magnification. **(B)** Renal cortex, 100× magnification. Chromatin condensation and nuclear shrinkage in proximal convolutes tubules (PCT) and distal convoluted tubules (DCT). **(C)** Outer stripe of the outer medulla (OSOM), 100× magnification. Chromatin condensation and nuclear shrinkage. **(D)** Inner stripe of the outer medulla (ISOM), 100× magnification. Chromatin condensation and nuclear shrinkage, cellular swelling and cytoplasmic dissolution, cellular rupture and sloughing. **(E)** Inner medulla, 100× magnification. No changes. **(F)** Pelvis and papilla, 100× magnification. No changes.

Four hours after death, the main postmortem findings were found in the tubules of the ISOM, where marked chromatin condensation and nuclear shrinkage of the epithelial cells and moderate cellular swelling and cytoplasmic dissolution with occasional cellular rupture was reported (Figure 4). Swelling was more prominent in the basal aspect of cells. Similar changes with less severity were also recorded in the DCT of the cortex (Figure 4B).

**Figure 5:**
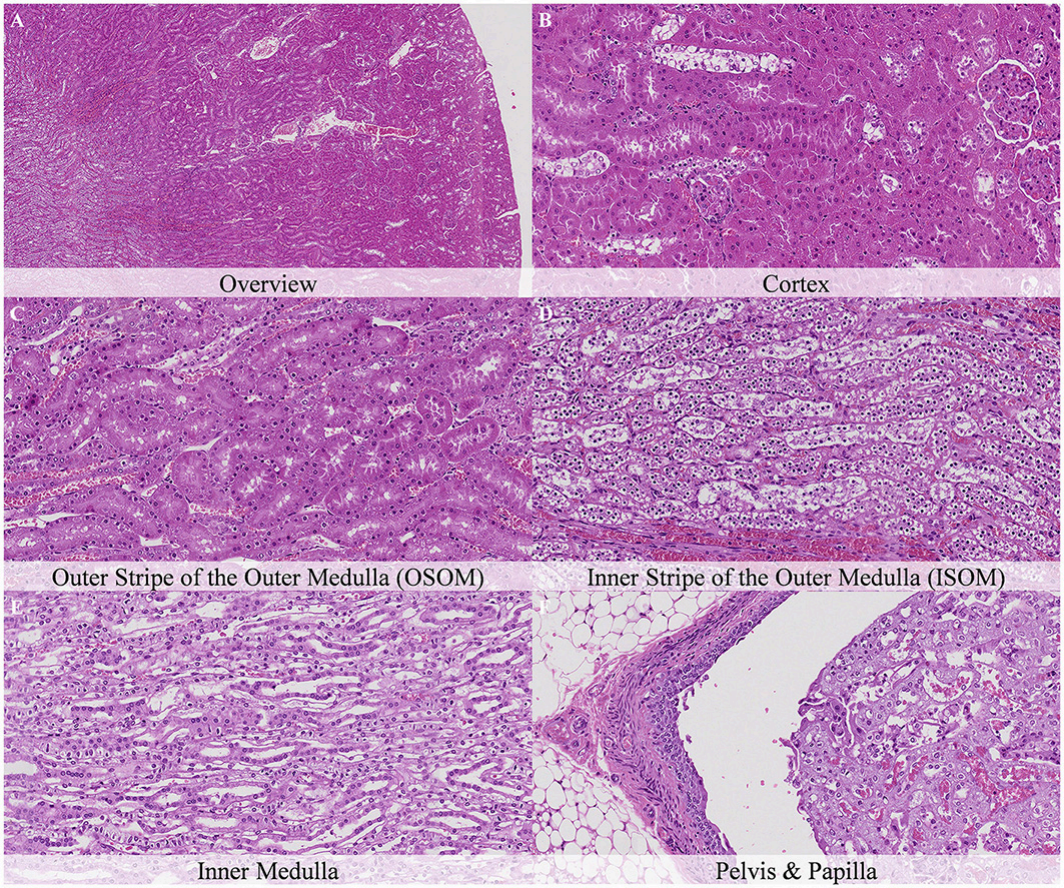
Postmortem microscopic changes in the kidney of non-exsanguinated outbred RccHan^TM^: WIST rats fixed 8 h after death. Carcass stored at room temperature (18–22°C). Hematoxylin-eosin stain. **(A)** Renal cortex, 20× magnification. **(B)** Renal cortex, 100× magnification. Chromatin condensation and nuclear shrinkage, cellular swelling and cytoplasmic dissolution in proximal convolutes tubules (PCT), and distal convoluted tubules (DCT), cellular rupture and sloughing in DCT. **(C)** Outer stripe of the outer medulla (OSOM), 100× magnification. Similar findings as noted after 4 h. **(D)** Inner stripe of the outer medulla (ISOM), 100× magnification. Similar findings as noted after 4 h, however, at higher severity. **(E)** Inner medulla, 100× magnification. Detachment from basement membrane. **(F)** Pelvis and papilla, 100× magnification. Urothelium detachment and sloughing.

Eight hours after death, cellular swelling and cytoplasmic dissolution of epithelial cells increased in severity in the DCT of the cortex and in the ISOM and started to be observed in the PCT of the cortex (Figure 5). In the DCT and in the ISOM this finding was associated with mild cellular rupture and sloughing. At this timepoint, the first morphological changes in the inner medulla were observed and they were characterized by mild cellular detachment from the basement membrane (Figure 5E). Similarly, the urothelium of the renal pelvis showed mild detachment and sloughing of the apical epithelial cell layers (Figure 5F).

**Figure 6:**
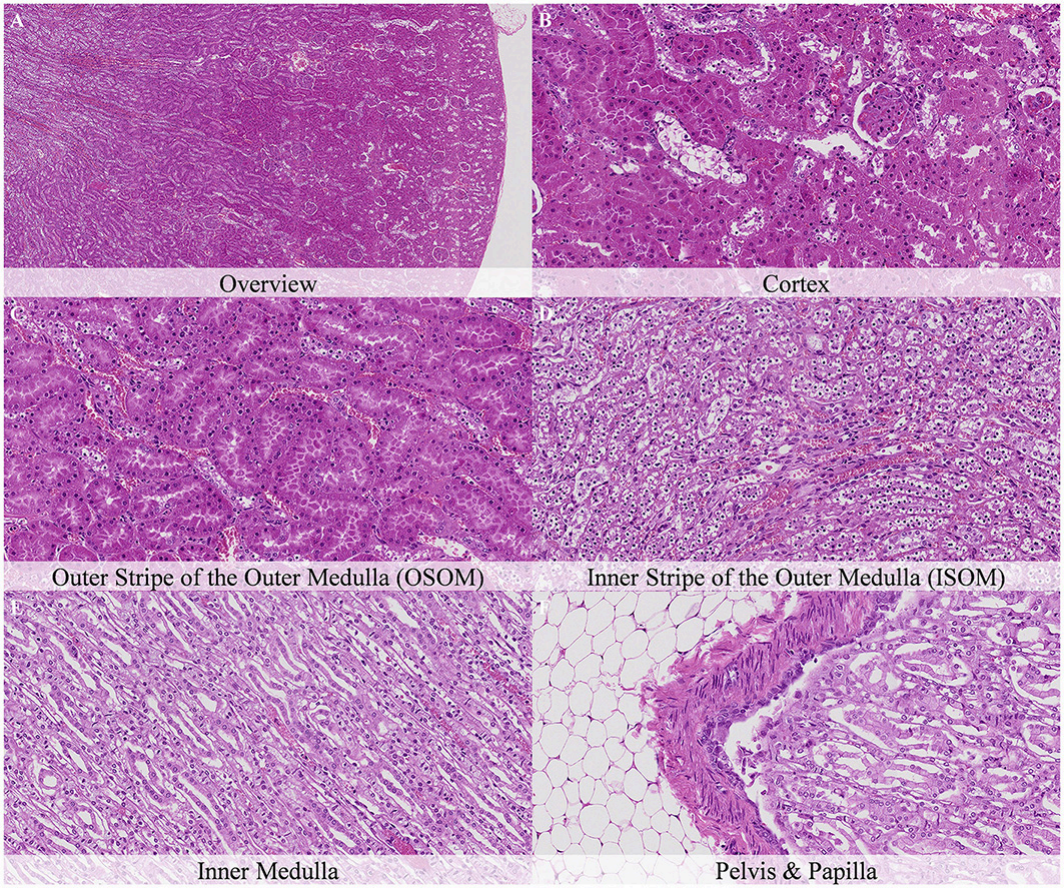
Postmortem microscopic changes in the kidney of non-exsanguinated outbred RccHan^TM^: WIST rats fixed 12 h after death. Carcass stored at room temperature (18–22°C). Hematoxylin-eosin stain. **(A)** Renal cortex, 20× magnification. **(B)** Renal cortex, 100× magnification. Glomerular changes start being present. **(C)** Outer stripe of the outer medulla (OSOM), 100× magnification. Incipient cellular swelling and cytoplasmic dissolution. **(D)** Inner stripe of the outer medulla (ISOM), 100× magnification. **(E)** Inner medulla, 100× magnification. **(F)** Pelvis and papilla, 100× magnification. Alterations similar in quality compared to those after 8 h, however, at a more pronounced severity.

Twelve hours after death, the first changes in glomeruli were reported, characterized by mild to moderate homogenization of the mesangium together with chromatin condensation and nuclear shrinkage of cells and presence of granular cell debris in the Bowman's space (Figure 6). Additionally, cellular swelling and cytoplasmic dissolution together with cellular rupture and sloughing increased in severity in the DCT (Figure 6B). The tubules in the OSOM showed moderate chromatin condensation and nuclear shrinkage and incipient cellular swelling and cytoplasmic dissolution (Figure 6C). Cellular detachment from the basal membrane in the tubules of the inner medulla and cellular rupture in PCT also increased in severity (Figure 6E).

**Figure 7:**
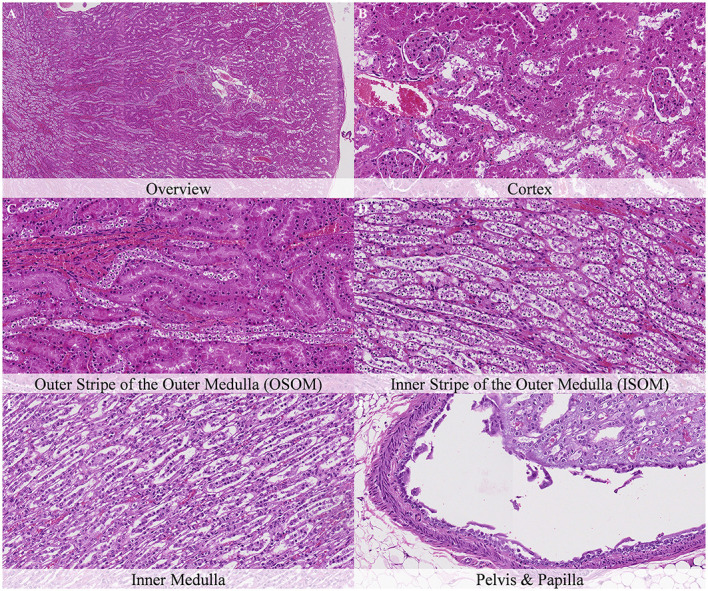
Postmortem microscopic changes in the kidney of non-exsanguinated outbred RccHan^TM^: WIST rats fixed 24 h after death. Carcass stored at room temperature (18–22°C). Hematoxylin-eosin stain. **(A)** Renal cortex, 20× magnification. **(B)** Renal cortex, 100× magnification. **(C)** Outer stripe of the outer medulla (OSOM), 100× magnification. **(D)** Inner stripe of the outer medulla (ISOM), 100× magnification. **(E)** Inner medulla, 100× magnification. Onset of cell shrinkage. **(F)** Pelvis and papilla, 100× magnification. Alterations similar in quality compared to those after 12 h, however, at a more pronounced severity. Cellular swelling and cytoplasmic dissolution are no longer recognizable in distal convoluted tubules (DCT).

Twenty-four hours after death, cellular swelling, cytoplasmic dissolution, cellular rupture, and sloughing increased in intensity in the PCT and the tubules in the ISOM and OSOM (Figure 7). However, cellular swelling and cytoplasmic dissolution could not be scored in the DCT due to the severe cellular rupture and sloughing reported in this structure (Figure 7B). In addition, moderate cellular detachment of the tubular epithelium of the inner medulla and urothelium of the renal pelvis was reported (Figures 7E, F). In the inner medulla, detached epithelium showed mild cellular shrinkage from this timepoint onwards (Figure 7E).

**Figure 8:**
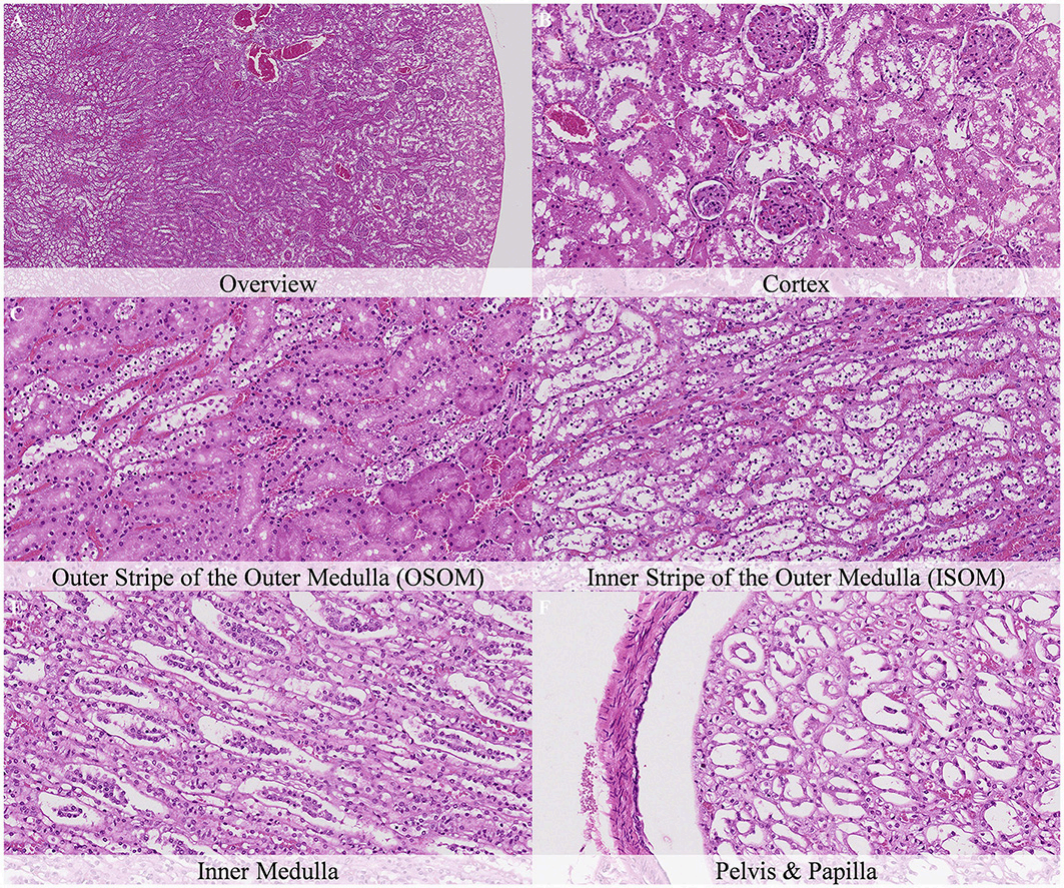
Postmortem microscopic changes in the kidney of non-exsanguinated outbred RccHan^TM^: WIST rats fixed 36 h after death. Carcass stored at room temperature (18–22°C). Hematoxylin-eosin stain. **(A)** Renal cortex, 20× magnification. **(B)** Renal cortex, 100× magnification. **(C)** Outer stripe of the outer medulla (OSOM), 100× magnification. **(D)** Inner stripe of the outer medulla (ISOM), 100× magnification. **(E)** Inner medulla, 100× magnification. **(F)** Pelvis and papilla, 100× magnification. Alterations similar in quality compared to those after 24 h, however, at a more pronounced severity.

Thirty-six hours after death, PCT showed marked chromatin condensation, nuclear shrinkage, cellular rupture, and sloughing associated with moderate coalescence of epithelial cells and multifocal loss of the basement membrane (Figure 8). Glomeruli and the tubules of the OSOM showed moderate chromatin condensation and nuclear shrinkage associated with nuclear fading (Figures 8C, D). The inner medulla showed increased severity of cellular shrinkage of the detached epithelium (Figure 8E).

**Figure 9:**
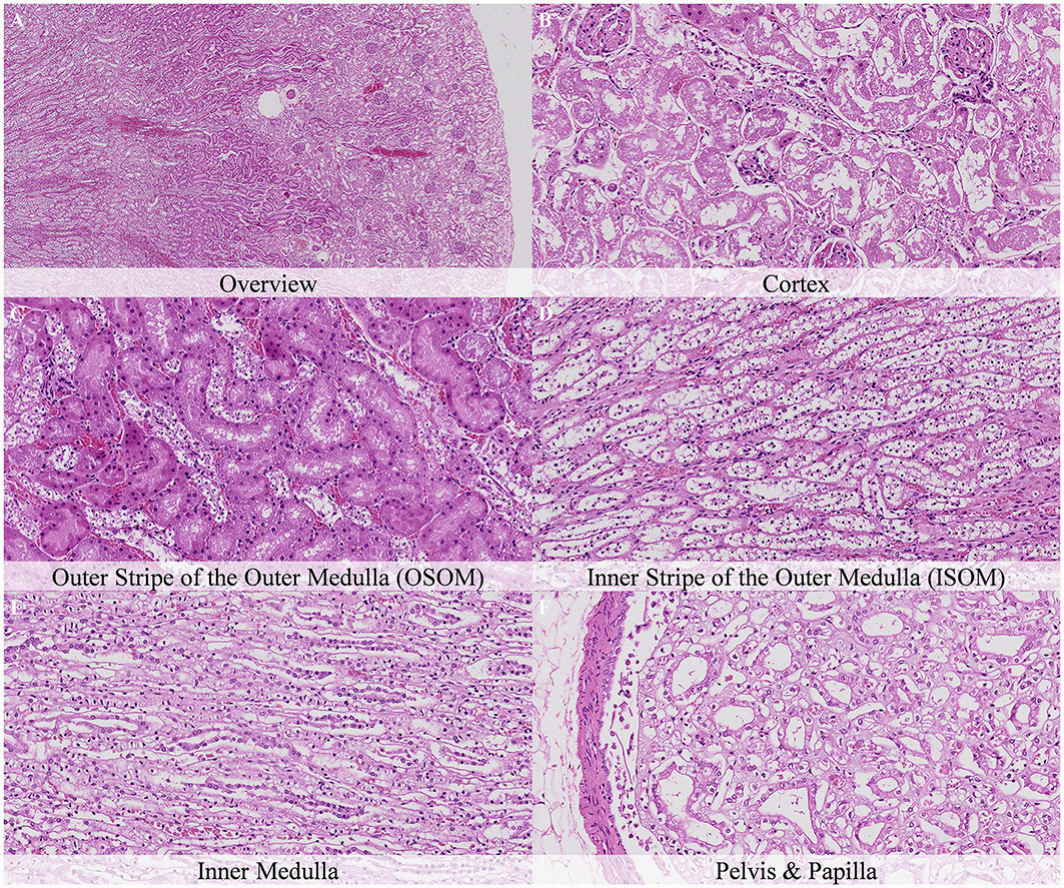
Postmortem microscopic changes in the kidney of non-exsanguinated outbred RccHan^TM^: WIST rats fixed 48 h after death. Carcass stored at room temperature (18–22°C). Hematoxylin-eosin stain. **(A)** Renal cortex, 20× magnification. **(B)** Renal cortex, 100× magnification. Proximal convoluted tubules (PCT) showed marked coalescence of epithelial cells. **(C)** Outer stripe of the outer medulla (OSOM), 100× magnification. **(D)** Inner stripe of the outer medulla (ISOM), 100× magnification. **(E)** Inner medulla, 100× magnification. **(F)** Pelvis and papilla, 100× magnification. Alterations similar in quality and severity compared to those after 36 h.

Forty-eight hours after death, PCT showed marked coalescence of epithelial cells that appeared as being partially ruptured and fused (Figure 9). Glomeruli showed protrusion of proximal tubules into the Bowman's space together with moderate amounts of granular cellular debris (Figure 9B).

### 3.3 Effect of exsanguination in rats stored at room temperature

**Figure 10:**
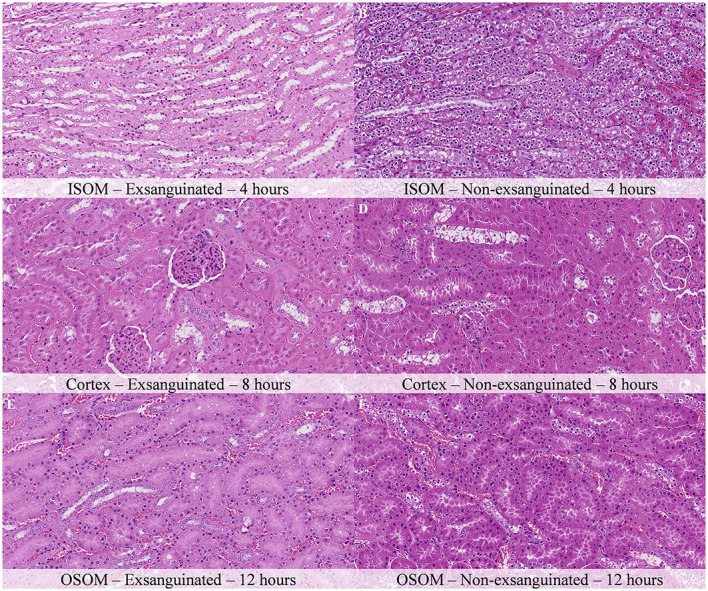
Comparison of postmortem histological changes in the kidney of exsanguinated and non-exsanguinated outbred RccHan^TM^: WIST rats after a delayed postmortem fixation. Carcass stored at room temperature (18–22°C). Hematoxylin-eosin stain. **(A, C, E)** Exsanguinated. **(B, D, F)** Non-exsanguinated. **(A, B)** Inner stripe of the outer medulla (ISOM), 4 h postmortem, 100× magnification. **(C, D)** Renal cortex, 8 h postmortem, 100× magnification. **(E, F)** Outer stripe of the outer medulla (OSOM), 12 h postmortem, 100× magnification. Postmortem changes progress faster in non-exsanguinated rats.

The nature of histopathology changes in animals exsanguinated immediately after death was identical to that in non-exsanguinated animals. The complete histological evaluation of these exsanguinated rats is displayed in Supplementary Table 2. Exsanguinated animals showed a moderate delay in the onset of postmortem changes and a slightly decreased severity of these changes at similar time points. For example, 4 h after death, exsanguinated animals showed mild chromatin condensation and nuclear shrinkage together with mild cellular swelling and cytoplasmic dissolution of tubular cells in the ISOM, which contrasts with the marked chromatin condensation and nuclear shrinkage, and the moderate cellular swelling and cytoplasmic dissolution of tubular cells recorded in non-exsanguinated kidneys (Figures 10A, B). Similar findings were observed in the DCT of the renal cortex 8 h after death, which showed chromatin condensation and nuclear shrinkage together with cellular swelling and cytoplasmic dissolution of mild severity in exsanguinated animals and of moderate severity in non-exsanguinated rats (Figures 10C, D). Twelve hours after death, chromatin condensation and nuclear shrinkage in the tubular epithelial cells of the OSOM showed also higher severity in non-exsanguinated animals (Figures 10E, F). Twenty-four hours after death, urothelial detachment and sloughing in the renal pelvis and epithelial cell detachment from the basement membrane in the inner medulla was more intense in non-exsanguinated animals than in exsanguinated ones.

### 3.4 Effect of cooling, air circulation, and body weight in non-exsanguinated rats

**Figure 11:**
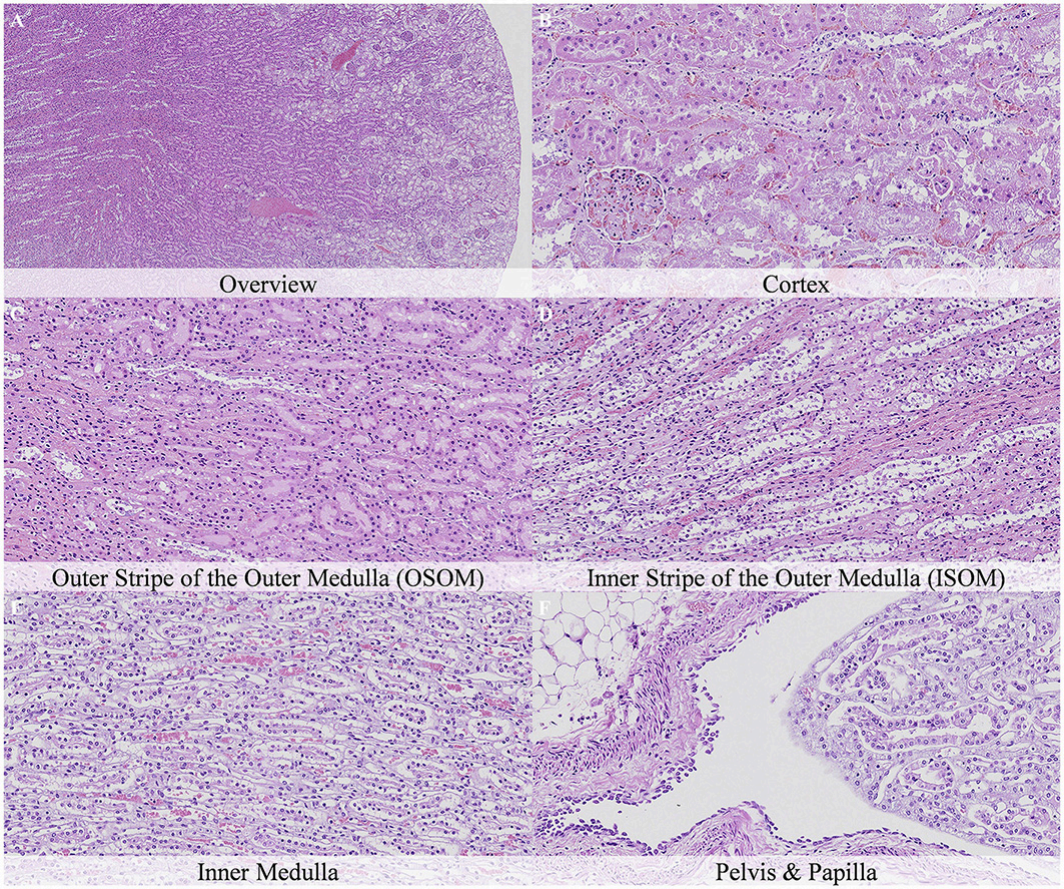
Postmortem microscopic changes in the kidney of non-exsanguinated outbred RccHan^TM^: WIST rats fixed 7 days after death. Carcass stored at room temperature (2–4°C). Hematoxylin-eosin stain. **(A)** Renal cortex, 20× magnification. **(B)** Renal cortex, 100× magnification. **(C)** Outer stripe of the outer medulla (OSOM), 100× magnification. **(D)** Inner stripe of the outer medulla (ISOM), 100× magnification. **(E)** Inner medulla, 100× magnification. **(F)** Pelvis and papilla, 100× magnification. Nature and intensity of postmortem changes similar to those recorded after 48 h at room temperature.

**Figure 12:**
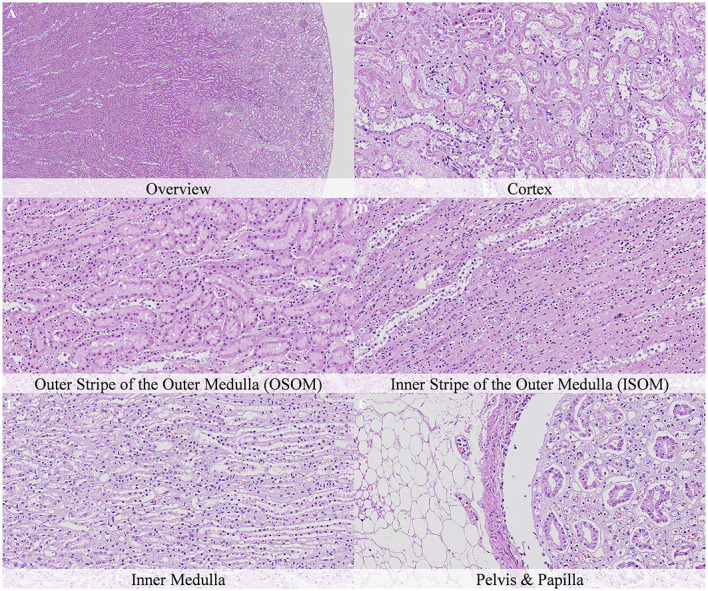
Postmortem microscopic changes in the kidney of non-exsanguinated outbred RccHan^TM^: WIST rats fixed 14 days after death. Carcass stored under refrigeration (2–4°C). Hematoxylin-eosin stain. **(A)** Renal cortex, 20× magnification. **(B)** Renal cortex, 100× magnification. **(C)** Outer stripe of the outer medulla (OSOM), 100× magnification. **(D)** Inner stripe of the outer medulla (ISOM), 100x magnification. **(E)** Inner medulla, 100x magnification. **(F)** Pelvis and papilla, 100× magnification. Nature and intensity of postmortem changes similar to those recorded after 48 h at room temperature.

Postmortem changes were recorded 7 and 14 days after death (Supplementary Table 3). The nature of the changes observed in animals stored under refrigeration was similar in animals stored at room temperature. In general, the intensity of postmortem changes observed after 7 and 14 days under refrigeration was similar to those recorded after 48 h at room temperature (Figures 11, 12). This finding demonstrates the positive effect of refrigeration in delaying the onset and severity of postmortem changes.

Although the weight of males was almost double than that of females, few differences were found in the severity of postmortem findings that could be attributed to animal weight. No clear differences were observed between animals stored in a closed plastic bag and animals stored in a perforated cardboard box to allow air circulation (Supplementary Table 3). Discrete differences were found in certain renal locations without a clear trend.

## 4 Discussion

The kidneys have a complex microanatomy and physiology to perform crucial metabolic functions such as the filtration of blood and the regulation of acid-base and electrolyte homeostasis. Autolysis, a well-known histological artifact associated with delayed fixation, frequently challenges pathologists in distinguishing these postmortem changes from genuine pathological renal findings, such as acute tubular injury (9, 10, 12, 13). Despite their importance, studies on postmortem microscopic renal changes are scarce, often covering only limited timepoints, focusing in other animal species, or displaying low amount of histopathology images to support toxicologic pathologists during their routine work (14–16).

This work focuses on the onset and progression of autolytic changes in the kidneys of Wistar Han rats, aiming to facilitate their identification and improve histopathological evaluations. The nomenclature proposed in the present work was further described and discussed on previous publications of our group (11). Briefly, the terminology aimed to be straightforward, descriptive, and consistent. It was customized among the 14 pathologists evaluating the slides, referring whenever possible to pre-existing terms already employed in the pathology literature and avoiding the use of specific diagnostic terms (10, 13, 17–22). Interestingly, some terms employed in the present work reflect histological features that are also found in physiological and pathological processes of cell degeneration and/or cell death (e.g., pyknosis is evidenced by chromatin condensation and nuclear shrinking) underscoring shared biochemical pathways between autolysis and *in vivo* degeneration (23–25).

The nature of histological changes was similar in all the animals of the study, with severity increasing over time, reinforcing the predictable, and progressive nature of postmortem alterations (2). Indeed, the present study provides a valuable framework for estimating postmortem intervals in spontaneous deaths occurring in regulatory and investigational studies with rodents. These results are potentially applicable to other species or humans, given the genetic homogeneity and controlled environments of laboratory rodents. However, variations in onset and progression across species are likely due to differences in body weight, cooling rates, and cellular metabolism (17, 21, 26, 27).

Postmortem changes differed among the different renal compartments. The cortex and the ISOM, containing the thin descending limb and the thick ascending limb of the Henle's loops, were the earliest and most affected compartments. These regions are considered to be highly susceptible to systemic injury due to the high metabolic oxygen demand and Na^+^K^+^ATPase activity of these tissues (28, 29). In the renal cortex, the DCT were more affected than the PCT at early timepoints. Cellular swelling and cytoplasmic dissolution was one of the main features in the DCT and evolved toward cellular rupture and sloughing. The higher intensity and progression of postmortem changes in the DCT in contrast to PCT may also be related with higher metabolic demands. On the one hand, loss of cellular ATP secondary to oxygen deprivation is the main driver of oncosis (a non-regulated form of cell death) due to alterations in the ionic balance and is considered the main pathway of postmortem cell death (22). On the other hand, oxidative stress has been shown to play a role in the onset of autolysis in renal tubules and Vitamin E deficiency has shown to accelerate the progression of postmortem changes in rats (30). Alternatively, the higher osmolarity of urine in the DCT could also play a role by triggering higher water influx by osmosis in the epithelial cells of these tubules in contrast to the PCT. Interestingly, renal tubules were affected earlier and more severely than glomeruli, consistent with prior reports emphasizing the diagnostic challenges in distinguishing autolysis from acute tubular damage (9, 10).

Host-related factors like exsanguination had a mild impact in the intensity of postmortem changes, with inefficient exsanguination potentially accelerating autolysis due to the release of substrates and enzymes from blood cells (28, 29, 31, 32). Previous studies highlight the role of animal size and weight in the progression of postmortem findings, particularly in larger species (33, 34). However, this could not be confirmed in the current study.

Regarding environmental conditions, refrigeration had a more pronounced impact on the rate of autolysis, slowing it significantly compared to room temperature storage, confirming its effectiveness in delaying postmortem changes (35–37). Contrarily, no clear differences were found in animals refrigerated in a closed plastic bag in comparison with animals refrigerated in a perforated cardboard box.

## 5 Conclusion

The present work provides a comprehensive assessment of the onset and progression of postmortem histological changes in the kidneys of rats, which are recognizable and evolve over time. Postmortem histopathological changes differ among the different renal anatomical compartments, likely associated with their cell composition and physiologic properties. Exsanguination and refrigeration of animals after death slow down the onset and progression of postmortem changes, whereas air circulation after death does not clearly have an impact.

## Data Availability

The original contributions presented in the study are included in the article/Supplementary material, further inquiries can be directed to the corresponding author.
