# Supplementary material for: Onset and progression of postmortem histological changes in the kidneys of RccHanTM:WIST rats
Source: Front Vet Sci. 2025 May 9;12:1578579. doi: 10.3389/fvets.2025.1578579 (PMC12100932; doi:10.3389/fvets.2025.1578579)
Supplement: Supplementary file 1 [file Data_Sheet_1.pdf]

Onset and Progression of Post-Mortem Histological Changes in the Kidneys of RccHan^{TM}:WIST Rats

Ricardo de Miguel[1*], Raquel Vallejo[1], Kristel Kegler[1], Robert Kreutzer[1], Francisco José Mayoral[2], Yoshimasa Okazaki[1], Paula Ortega[1], Laura Polledo[1], Tanja Razinger[1], Olivia Kristina Richard[1], Raúl Sanchez[2], Nils Warfving[1], Anna Domènech[2], Klaus Weber[1].

[1]AnaPath Services GmbH, Liestal, Switzerland.

[2]AnaPath Research S.A.U., Santa Perpètua de Mogoda, Barcelona, Spain

SUPPLEMENTARY TABLES:

Supplementary Table S1: Timepoints and conditions evaluated during the study.

Supplementary Table S2: Postmortem histological findings in the kidney of exsanguinated outbred RccHan^{TM}: WIST rats stored at room temperature (18-22 ºC) and necropsied at different time points after death. Postmortem changes were scored from 0 to 3 (i.e., 0 = absent to minimal; 1 = mild; 2 = moderate; 3 = marked) based on their distribution, extension, and intensity degree.

Supplementary Table 3: Postmortem histological findings in the kidney of non-exsanguinated outbred RccHanTM: WIST rats stored under refrigeration (2-4 ºC) and necropsied at different time points after death. Post-mortem changes were scored from 0 to 3 (i.e., 0 = absent to minimal; 1 = mild; 2 = moderate; 3 = marked) based on their distribution, extension, and intensity degree.

Supplementary Table 1: Timepoints and conditions evaluated during the study.

Temperature	Postmortem time	Sex	Exsanguination status	Storage
Room temperature (18-22ºC)	0.5h	Female	Exsanguinated	Uncovered
			Non-exsanguinated	Uncovered
	1h	Female	Exsanguinated	Uncovered
			Non-exsanguinated	Uncovered
	4h	Female	Exsanguinated	Uncovered
			Non-exsanguinated	Uncovered
	8h	Female	Exsanguinated	Uncovered
			Non-exsanguinated	Uncovered
	12h	Female	Exsanguinated	Uncovered
			Non-exsanguinated	Uncovered
	24h	Female	Exsanguinated	Uncovered
			Non-exsanguinated	Uncovered
	36h	Female	Exsanguinated	Uncovered
			Non-exsanguinated	Uncovered
	48h	Female	Exsanguinated	Uncovered
			Non-exsanguinated	Uncovered
Refrigerator (2-4ºC)	7d	Male	Non-exsanguinated	Plastic bag
				Cardboard box
		Female	Non-exsanguinated	Plastic bag
				Cardboard box
	14d	Male	Non-exsanguinated	Plastic bag
				Cardboard box
		Female	Non-exsanguinated	Plastic bag
				Cardboard box

Supplementary Table 2: Postmortem histological findings in the kidney of exsanguinated outbred RccHan^{TM}: WIST rats stored at room temperature (18-22 ºC) and necropsied at different time points after death. Postmortem changes were scored from 0 to 3 (i.e., 0 = absent to minimal; 1 = mild; 2 = moderate; 3 = marked) based on their distribution, extension, and intensity degree.

	**TIME AFTER DEATH**							
	**0.5h**	**1h**	**4h**	**8h**	**12h**	**24h**	**36h**	**48h**
**CORTEX - Glomeruli**								
Chromatin condensation & Nuclear shrinkage	-	-	-	-	1	1	2 [a]	2 [a]
Homogenisation of mesangium	-	-	-	-	1	2	2	2 [b]
Granular Cellular debris	-	-	-	-	1	1	2	1
AVERAGE	0.00	0.00	0.00	0.00	1.00	1.33	2.00	1.67
**CORTEX – Proximal Convoluted Tubule (PCT)**								
Chromatin condensation & Nuclear shrinkage	-	-	1	1	1[a]	2[a]	3[a]	2[a]
Cellular swelling &Cytoplasmic dissolution	-	-	-	1	1	2	N/Q	N/Q
Cellular rupture & Sloughing	-	-	-	-	1[c]	2[c]	3[c]	3[c]
Coalescence of Epithelia	-	-	-	-	-	-	2	2
AVERAGE	0.00	0.00	0.25	0.50	0.75	1.50	2.75	2.50
**CORTEX – Distal Convoluted Tubule (DCT)**								
Chromatin condensation & Nuclear shrinkage	-	-	1	1	2	3	3	3
Cellular swelling & Cytopl. dissolution	-	1	1	1	2	3	N/Q	N/Q
Cellular rupture & Sloughing	-	-	-	1	1	3	3	3
AVERAGE	0.00	0.33	0.67	1.00	1.67	3.00	3.00	3.00
**OUTER STRIPE OUTER MEDULLA (OSOM)**								
Chromatin condensation & Nuclear shrinkage	-	-	1	1	1	1	2[a]	2[a]
Cellular swelling & Cytopl. dissolution	-	-	-	-	1	2	3	3
AVERAGE	0.00	0.00	0.50	0.50	1.00	1.50	2.50	2.50
**INNER STRIPE OUTER MEDULLA (ISOM)**								
Chromatin condensation & Nuclear shrinkage	-	-	1	3	3	3	3	3
Cellular swelling & Cytopl. dissolution[d]	-	-	1[d]	2[d]	3[d]	3[d]	2	3
Cellular rupture & Sloughing	-	-	-	1	1	2	3	3
AVERAGE	0.00	0.00	1.00	2.00	2.33	2.67	2.67	3.00
**INNER MEDULLA**								
Cellular Detachment	-	-	-	-	1	3	2	3
Cellular shrinkage	-	-	-	-	-	2	1	2
AVERAGE	0.00	0.00	0.00	0.00	1.00	2.50	1.50	2.50
**PELVIS & PAPILLA**								
Urothelium detachment/sloughing	-	-	-	1	1	1	3	3

[a] Ass. with nuclear fading

[b] Protrusion of proximal tubule

[c] Ass. with lumen debris

[d] More pronounced basally

Supplementary Table 3: Postmortem histological findings in the kidney of non-exsanguinated outbred RccHanTM: WIST rats stored under refrigeration (2-4 ºC) and necropsied at different time points after death. Post-mortem changes were scored from 0 to 3 (i.e., 0 = absent to minimal; 1 = mild; 2 = moderate; 3 = marked) based on their distribution, extension, and intensity degree.

	PLASTIC BAG				CARDBOARD BOX			
	7 days		14 days		7 days		14 days	
	F	M	F	M	F	M	F	M
**CORTEX - Glomeruli**								
Chromatin condensation & Nuclear shrinkage	2 [a]	2 [a]	2 [a]	2 [a]	2 [a]	2 [a]	2 [a]	2 [a]
Homogenisation of mesangium	2[b]	2[b]	2[b]	2[b]	2[b]	2[b]	2[b]	2[b]
Granular Cellular debris	1	1	1	1	1	1	1	1
AVERAGE	1.67	1.67	1.67	1.67	1.67	1.67	1.67	1.67
**CORTEX – Proximal Convoluted Tubule (PCT)**								
Chromatin condensation & Nuclear shrinkage	3[a]	3[a]	3[a]	3[a]	3[a]	3[a]	3[a]	3[a]
Cellular swelling &Cytoplasmic dissolution	N/Q	N/Q	N/Q	N/Q	N/Q	N/Q	N/Q	N/Q
Cellular rupture & Sloughing	3[c]	3[c]	3[c]	3[c]	3[c]	3[c]	3[c]	3[c]
Coalescence of Epithelia	3	3	3	3	3	3	3	3
AVERAGE	3.00	3.00	3.00	3.00	3.00	3.00	3.00	3.00
**CORTEX – Distal Convoluted Tubule (DCT)**								
Chromatin condensation & Nuclear shrinkage	3	3	3	3	3	3	3	3
Cellular swelling & Cytopl. dissolution	N/Q	N/Q	N/Q	N/Q	N/Q	N/Q	N/Q	N/Q
Cellular rupture & Sloughing	3	3	3	3	3	3	3	3
AVERAGE	3.00	3.00	3.00	3.00	3.00	3.00	3.00	3.00
**OUTER STRIPE OUTER MEDULLA (OSOM)**								
Chromatin condensation & Nuclear shrinkage	2[a]	2[a]	2[a]	2[a]	2[a]	2[a]	2[a]	2[a]
Cellular swelling & Cytopl. dissolution	3	3	3	3	3	3	3	3
AVERAGE	2.50	2.50	2.50	2.50	2.50	2.50	2.50	2.50
**INNER STRIPE OUTER MEDULLA (ISOM)**								
Chromatin condensation & Nuclear shrinkage	3	3	3	3	3	3	3	3
Cellular swelling & Cytopl. dissolution	3	3	3	3	3	3	3	3
Cellular rupture & Sloughing	3	3	3	3	3	3	3	3
AVERAGE	3.00	3.00	3.00	3.00	3.00	3.00	3.00	3.00
**INNER MEDULLA**								
Cellular Detachment	3	3	3	3	3	3	3	3
Cellular shrinkage	2	2	2	2	2	2	2	2
AVERAGE	2.50	2.50	2.50	2.50	2.50	2.50	2.50	2.50
**PELVIS & PAPILLA**								
Urothelium detachment/sloughing	3	3	3	3	3	3	3	3

[a] Ass. with nuclear fading

[b] Protrusion of proximal tubule

[c] Ass. with lumen debris
